# Structural and Molecular Conservation of Glucagon-Like Peptide-1 and Its Receptor Confers Selective Ligand-Receptor Interaction

**DOI:** 10.3389/fendo.2012.00141

**Published:** 2012-11-19

**Authors:** Mi Jin Moon, Sumi Park, Dong-Kyu Kim, Eun Bee Cho, Jong-Ik Hwang, Hubert Vaudry, Jae Young Seong

**Affiliations:** ^1^Graduate School of Medicine, Korea UniversitySeoul, Republic of Korea; ^2^INSERM U982, Laboratory of Neuronal and Neuroendocrine Differentiation and Communication, University of RouenMont-Saint-Aignan, France

**Keywords:** GLP-1, GLP1R, G protein-coupled receptors, evolution, paralog, ortholog, ligand-receptor interaction

## Abstract

Glucagon-like peptide-1 (GLP-1) is a major player in the regulation of glucose homeostasis. It acts on pancreatic beta cells to stimulate insulin secretion and on the brain to inhibit appetite. Thus, it may be a promising therapeutic agent for the treatment of type 2 diabetes mellitus and obesity. Despite the physiological and clinical importance of GLP-1, molecular interaction with the GLP-1 receptor (GLP1R) is not well understood. Particularly, the specific amino acid residues within the transmembrane helices and extracellular loops of the receptor that may confer ligand-induced receptor activation have been poorly investigated. Amino acid sequence comparisons of GLP-1 and GLP1R with their orthologs and paralogs in vertebrates, combined with biochemical approaches, are useful to determine which amino acid residues in the peptide and the receptor confer selective ligand-receptor interaction. This article reviews how the molecular evolution of GLP-1 and GLP1R contributes to the selective interaction between this ligand-receptor pair, providing critical clues for the development of potent agonists for the treatment of diabetes mellitus and obesity.

## Introduction

Glucagon-like peptide-1 (GLP-1) is an intestinal incretin released in response to nutrient ingestion that stimulates insulin secretion in a glucose-dependent manner. The insulinotropic effect of GLP-1 on the pancreas has been demonstrated to be preserved in animal models of diabetes by stimulating insulin exocytosis (Shen et al., 1998; Drucker, 2001; MacDonald et al., 2002), and promoting insulin biosynthesis (Fehmann and Habener, 1992; Perfetti and Merkel, 2000; Moon et al., 2011). Recently, direct effects of GLP-1 on growth, survival (Xu et al., 1999; Stoffers et al., 2000; List and Habener, 2004), and differentiation of β-cells have been reported (Drucker, 2003). Beside its insulinotropic effects, GLP-1 inhibits glucagon (GCG) secretion in pancreatic α-cells (Nauck et al., 2002), attenuates gastric emptying, and ameliorates glucose excursion in the gastrointestinal tract (Nauck et al., 2002).

GLP-1 exerts its action through the G protein-coupled receptor (GPCR), GLP1R. This receptor belongs to the class B (or secretin-like) GPCR family. This GPCR family has only 15 members in humans and is characterized by a relatively long N-terminal extracellular domain (ECD) containing six conserved Cys residues that form a constraint structure necessary for ligand binding (Couvineau et al., 2004). GLP1R is mainly expressed in pancreatic β-cells, and upon stimulation by GLP-1, it induces the accumulation of cAMP and the influx of intracellular calcium which accelerate insulin release from secretory granules (Drucker et al., 1987; Fehmann et al., 1995).

GLP-1 and GLP1R are also expressed in the central nervous system. GLP-1 is synthesized largely in the brainstem and transported along axonal networks to diverse brain regions, including the hypothalamus (Vrang et al., 2007; Hisadome et al., 2010). GLP1R is expressed in cerebral cortex, hypothalamus, hippocampus, thalamus, caudate-putamen, and globus pallidum (Alvarez et al., 2005). In the brain, GLP-1 is known to reduce appetite, leading to significant reductions in body weight (Zander et al., 2002). In addition, GLP-1 is likely neuroprotective and involved in neurite growth and spatial learning ability in the brain (During et al., 2003; Perry et al., 2007).

Due to its combined beneficial effects, GLP-1 has been identified as a potential therapeutic agent for the treatment of diabetes mellitus and obesity. However, the molecular mechanisms leading to high affinity ligand-receptor binding and receptor activation have not been fully understood. Studies using alanine scanning mutagenesis, substitution/modification, and chimeric peptide construction of GLP-1 have explored the bioactive motifs of GLP-1(Adelhorst et al., 1994; Gallwitz et al., 1994; Hinke et al., 2004), yet none have been able to identify the mechanism through which individual residues in the peptide interact with residues in the receptor. Recent studies using X-ray crystallography have demonstrated that residues in the central α-helical region of GLP-1 interact with residues in the N-terminal ECD of GLP1R (Runge et al., 2008; Underwood et al., 2010). However, as ligand-induced receptor activation is mainly governed by interactions between residues in the N-terminus of the peptide and residues within the transmembrane helices (TMH) and extracellular loops (ECL) of the receptor (Thorens et al., 1993; During et al., 2003), this crystal structure only accounts for the peptide binding to the ECD of the receptor. Indeed, virtually no progress has been made in exploring the receptor binding sites for the N-terminal moiety of the peptide which is responsible for ligand-induced receptor activation.

Peptide ligands and their receptors have become diversified through evolutionary processes that ultimately yield families of related, yet distinct, peptides and receptors (Hoyle, 1999; Cho et al., 2007; Lee et al., 2009; Kim et al., 2011, 2012). Specific diversification of peptides, conservation within orthologs but variation among paralogs, would often confer the selective interaction with their cognate receptors, allowing discrimination of paralogous receptors (Acharjee et al., 2004; Wang et al., 2004; Li et al., 2005). Thus, amino acid sequence comparison of the peptide and receptor with their orthologs and paralogs, along with mutational mapping approaches, are useful tools helping to determine specific residues in the peptide ligands and receptors that are essential for maintaining selective ligand-receptor interaction. Indeed, ligand binding domains identified in mammalian receptors are highly conserved in orthologous non-mammalian receptors, indicating that there is high evolutionary selection pressure to maintain selectivity for their ligands (Acharjee et al., 2004; Wang et al., 2004; Li et al., 2005). Recently, we reported that evolutionarily conserved amino acid residues in GLP-1 and core domains of the GLP1R confer selective ligand-receptor interaction and receptor activation (Moon et al., 2010, 2012). This article reviews how the molecular evolution of GLP-1 and GLP1R contributes to acquiring high affinity interaction between this peptide ligand and receptor.

## General Structure of GLP-1 and Its Family Peptides

GLP-1 is a product of the *GCG* gene which encodes a common GCG-GLP-1-GLP-2 precursor. All three peptides are encoded by different exons of the *GCG* gene, raising the possibility of exon duplications during early vertebrate evolution (Sherwood et al., 2000). The *GCG* gene produces one or two mature peptides by a tissue-specific alternative post-translational process (Kieffer and Habener, 1999; Irwin, 2001, 2009). For instance, in pancreatic α-cells mature GCG, but not GLP-1 and GLP-2, is produced. In intestinal L cells, however, mature GLP-1 and GLP-2, but not GCG, are generated. One GLP-1 paralog is the glucose-dependent insulinotropic polypeptide (GIP) which is independently encoded by the *GIP* gene (McIntosh et al., 2009). GLP-1 and GIP share a high degree of amino acid sequence identity, particularly in their N-terminal moiety, and function similarly by inducing insulin secretion from β-cells. However, these peptides act through distinct yet related receptors, GLP1R and GIP receptor (GIPR), respectively. In addition, albeit absent in mammals, another GLP-1 paralog is exendin, which was first discovered in Gila monsters (*Heloderma suspectum*; Göke et al., 1993). Recently, the full-length and/or partial cDNAs for exendin were characterized in a few species, such as *Xenopus*, chicken, and Gila monster (Irwin and Prentice, 2011). Although the receptor for exendin has not yet been identified in non-mammals, exendin exhibits high affinity binding for mammalian GLP1R (Göke et al., 1993). Recent evidence suggests that the *GCG*, *GIP*, and *exendin* genes were generated by genome duplication events during early vertebrate evolution, as these genes are flanked by similar neighboring genes in the genomes of vertebrates (Irwin, 2002; Irwin and Prentice, 2011).

GLP-1 and its family peptides are 30 ∼ 40 amino acids in length and share similarities in amino acid sequence and secondary structure. All these peptides tend to be disordered in aqueous solutions but exhibit a marked propensity to form α-helices under mild ambient conditions, such as in the presence of organic solvents or lipids (Braun et al., 1983; Gronenborn et al., 1987; Thornton and Gorenstein, 1994; Inooka et al., 2001; Neidigh et al., 2001; Chang et al., 2002; Tan et al., 2006; Alana et al., 2007), or upon crystallization (Sasaki et al., 1975). It is now well known that the N-terminal domains of GLP-1-related peptides form a random coil structure, while the central parts of these peptides have an α-helical structure. In addition, hydrophobic amino acids at positions 6 and 10, and a short-chain polar amino acid at position 7 form a helix N-capping motif through hydrophobic interaction and hydrogen bonding (Neumann et al., 2008). This capping motif is believed to introduce a specific local fold that facilitates receptor activation upon peptide-receptor binding (Neumann et al., 2008).

**Figure 1:**
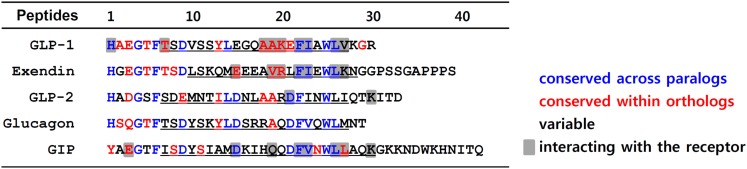
**Amino acid sequence alignment of GLP-1 and its family peptides**. Amino acid sequences of human GLP-1, GLP-2, glucagon, GIP, and Gila monster exendin are aligned. Residues in red are conserved sequences within orthologs of vertebrates including mouse, anole, chicken, *Xenopus tropicalis*, medaka, fugu, tetraodon, stickleback, and zebrafish. The residues colored in blue represent conserved sequences across paralogs. The residues in black are variable sequences. Underlines indicate the α-helical conformation. The residues responsible for interaction with their receptor are shaded.

The N-terminus of GLP-1 and its family peptides share a high degree of sequence identity (Figure 1). Particularly, Gly^4^, Thr/Ser^5^, Phe^6^, and Asp/Glu^9^ are conserved across all GLP-1 paralogs. Indeed, alanine scanning of these conserved residues of GLP-1 suggest that positions 4, 6, and 9 are crucial for either maintaining secondary structure of the peptide or for interaction with the receptor (Adelhorst et al., 1994; Gallwitz et al., 1994). His^1^ and Thr/Ser^7^ are common for most GLP-1 paralogs except for GIP, which has Tyr^1^ and Ile^7^ in these positions. Our recent observation using a chimeric GLP-1/GIP peptide revealed that His/Tyr^1^ and Thr/Ile^7^ are responsible for the selective interaction toward GLP1R and GIPR (Moon et al., 2010). The second position of the peptides is highly variable across paralogs, even within orthologs of vertebrates, and it is known to be of lesser importance for receptor binding. This residue can be modified to Ser or another amino acid to confer protection against cleavage by dipeptidyl peptidase IV (Hinke et al., 2002). The third position of the peptides is variable across paralogs but conserved within orthologs. Although the importance of this ortholog-specific third residue in receptor binding or peptide structure is not fully understood, Glu^3^ of GIP is known to be critical for receptor interaction (Hinke et al., 2003; Gault et al., 2007; Yaqub et al., 2010).

The sequence similarity of the α-helix domain of the peptides is not pronounced among GLP-1 paralogs, and there are also many variable residues within orthologs (Figure 1). However, the Phe^22^, Ile/Val^23^, and Leu^26^ residues that are part of the hydrophobic surface of the α-helix are highly conserved among GLP-1 and its paralogs. Interestingly, all these residues are known to interact with highly conserved residues in the ECD of the GLP1R and its paralogs (Parthier et al., 2007; Runge et al., 2008; Underwood et al., 2010). Some ortholog-conserved residues, Glu/Asp^15^ of exendin and GIP, along with Ala^18/19^ and Lys^20^ of GLP-1, are found to interact with the receptors as was revealed by the peptide-bound ECD crystal structures (Parthier et al., 2007; Runge et al., 2008; Underwood et al., 2010).

## General Structure of Class B GPCRs

The class B GPCR family is composed of 15 members including receptors for VIP, PACAP, secretin, GCG, GLP-1, GLP-2, GHRH, GIP, PTH, calcitonin, calcitonin gene-related peptide, and CRH (Laburthe et al., 2007). This family shares the general GPCR architecture: seven TMH interconnected by intracellular loops with a C-terminal intracellular domain. Class B GPCRs differ from class A rhodopsin-like GPCRs in the structures of their TMH. The TMH of class B GPCRs do not contain conserved amino acid residues such as Asp/Asn^2.50^ in TMH2, Asn/Asp^7.49^-Pro^7.50^-x-x-Tyr^7.53^ (N/DPxxY) motif in TMH7, Asp/Glu^3.49^-Arg^3.50^-Tyr/Trp^3.51^ (D/ERY/W) motif at the junction between TMH3 and intracellular loop 2 that are commonly found in class A rhodopsin-like GPCRs (Oh et al., 2005). Instead, class B receptors share a high degree of amino acid identity in TMH with one another. Further, they possess a large and structured N-terminal ECD of ∼120 residues.

Although no experimentally determined full-length class B receptor structure has been achieved to date, the structure elucidation of individual class B GPCR ECDs represents considerable progress toward a molecular understanding of their action. The first structure of the agonist-bound recombinant N-terminal ECD of the CRH2B receptor has been resolved using NMR (Grace et al., 2004). Six representative ECD structures of the class B family of GPCRs have been determined by X-ray crystallography or NMR spectroscopy in complex with bound ligand: the human VPAC1 receptor (Tan et al., 2006), a subtype of human PACAP receptor (PAC1R_s_; Sun et al., 2007), human GIPR (Parthier et al., 2007), human GLP1R (Runge et al., 2008; Underwood et al., 2010), human PTH receptor (PTH1R; Pioszak and Xu, 2008), the human type-1 CRF receptor (CRFR1; Pioszak et al., 2008), and human GLP2R (Venneti and Hewage, 2011).

**Figure 2:**
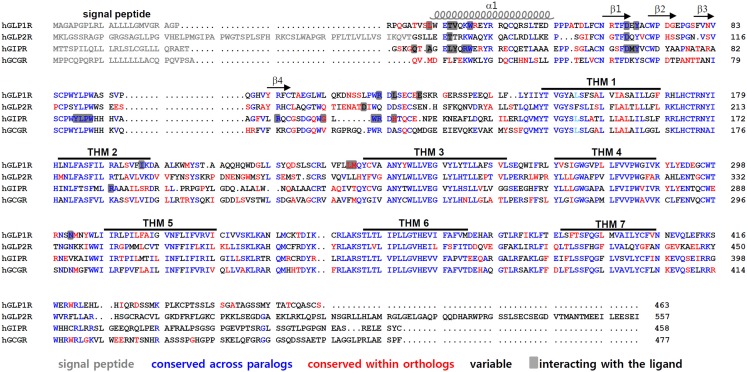
**Amino acid sequence alignment of GLP1R and its paralogous receptors**. Amino acid sequences of human GLP1R, GLP2R, GCGR, and GIPR are compared. The signal peptides are indicated in gray. The residues colored in blue represent conserved sequences across the GLP-1-related peptide receptors. Residues in red are conserved sequences within orthologs of vertebrates such as mouse, anole, chicken, *Xenopus tropicalis*, medaka, fugu, tetraodon, stickleback, and zebrafish. The residues in black are variable sequences. The amino acid residues that interact with their peptides are shaded. The α-helix, β-sheets, and TMH domains of GLP1R are indicated.

Class B GPCRs contain N-terminal signal peptides that are cleaved off by the signal peptidase of the endoplasmic reticulum (ER) during the translocation-mediated receptor insertion into the ER membrane. These signal peptides play a crucial role in the membrane expression of receptors (Couvineau et al., 2004; Alken et al., 2005; Huang et al., 2010). After cleavage of the signal peptide, the N-terminal helix at the beginning of the ECD and four β-strands forming two antiparallel sheets remain (Figure 2). Three disulfide bonds formed by a set of six Cys residues lock these secondary structural elements together. Cysteine residues are completely conserved across the receptors. The disulfide bond pattern seems to be conserved in all receptors, suggesting a similar three dimensional structure. There are three disulfide bonds between the first and third, the second and fifth, and the fourth and sixth cysteine residues (Cys^1^-Cys^3^, Cys^2^-Cys^5^, Cys^4^-Cys^6^). The first bond (Cys^1^-Cys^3^) links the N-terminal α-helix to the first β-sheet. The second (Cys^2^-Cys^5^) connects the two β-sheets, whereas the third disulfide bond (Cys^4^-Cys^6^) holds the C-terminus of the domain in close proximity to the central β-sheets (Tan et al., 2006; Parthier et al., 2007, 2009; Sun et al., 2007; Pioszak and Xu, 2008; Pioszak et al., 2008; Runge et al., 2008; Underwood et al., 2010; Venneti and Hewage, 2011).

In addition, this core folding is further stabilized by a salt bridge involving acidic and basic residues flanked by hydrophobic aromatic residues. This fold, called the Sushi domain, is conserved in all class B GPCRs (Grace et al., 2004). Five additional residues, Asp^67^, Trp^72^, Pro^86^, Gly^108^, and Trp^110^ in GLP1R and the corresponding residues in class B GPCRs, are conserved. Particularly, Asp and the two Trp residues take part in forming the Sushi domain, suggesting that these residues are crucial for domain stability and ligand binding. The strongly conserved fold observed in the ECD of class B GPCRs suggests that a common mechanism underlies ligand recognition.

The crystal structures of the ligand-bound ECD reveal amino acid residues that interact with their cognate ligands (Parthier et al., 2007; Runge et al., 2008; Underwood et al., 2010). It is of interest to note that many ligand-interacting residues are highly conserved between GLP1R and its paralogs. For instance, Trp^39^ in the α1-helix, Asp^67^ in the β1-sheet, Tyr^69^ between the β1- and β2-sheet, Arg^121^ near TMH1 of GLP1R, residues 87–90 (Tyr-Leu-Pro-Trp) between the β3- and β4-sheet, Arg^101^ in the β4-sheet, and Trp^112^ near TMH1 of GIPR are all highly conserved across paralogs (Figure 2). There are also ortholog-specific residues that interact with the peptide ligands, such as Leu^32^ in the α1-helix of GLP1R, and Gly^110^ and His^115^ near TMH1 of GIPR.

## The Two-domain Hypothesis for Class B GPCR Ligand Binding and Activation

Class B GPCRs likely share a similar secondary and tertiary structure with long N-terminal ECD and highly conserved TMHs. The orientation and mechanism of interaction of the peptide with their receptors has been investigated in studies using fragmented peptide/receptor, and chimeric peptides and receptors (Holtmann et al., 1995; Stroop et al., 1995; Bergwitz et al., 1996; Laburthe and Couvineau, 2002; Runge et al., 2003). For instance, the GLP1R ECD itself is able to bind with its peptide ligands (Graziano et al., 1996; Van Eyll et al., 1996; Wilmen et al., 1996, 1997; Runge et al., 2003; Parthier et al., 2007). This binding, however, may not account for ligand-induced receptor activation (Buggy et al., 1995; Holtmann et al., 1995, 1996; Hjorth and Schwartz, 1996; Xiao et al., 2000). The N-terminally truncated exendin(9–39) is unable to activate GLP1R even though it binds to the receptor with an affinity that is comparable to that of wild type exendin (Thorens et al., 1993). In contrast, exendin(1–9), a short N-terminal fragment of exendin, is able to activate GLP1R although its affinity to the receptor is quite low (During et al., 2003). Likewise, the GIP fragment, GIP (7–30), is able to bind to GIPR with high affinity but fails to induce receptor activation. In contrast, GIP (1–14) exhibits a very low affinity toward the receptor but fully activates the receptor at a micromolar concentration (Hinke et al., 2001, 2003; Gault et al., 2007). Thus, the two-domain model explaining ligand binding followed by receptor activation has emerged: the central α-helical and C-terminal portion of the peptide binds to the N-terminal ECD of the receptor (Al-Sabah and Donnelly, 2003; Dong et al., 2003; Lopez de Maturana et al., 2003) followed by binding of the N-terminal moiety of the peptide with the core domain – including the TMH and ECL – of the receptor, conferring receptor activation and G protein coupling (Runge et al., 2003; Lopez de Maturana et al., 2004; Castro et al., 2005; Wittelsberger et al., 2006). The two-domain model is generally consistent with photoaffinity crosslinking studies of several class B receptors. With a few exceptions, photoreactive side chains in the C-terminus of the peptide ligand interact with residues in the ECD of the receptor, whereas photoreactive side chains in the N-terminus of the ligand bind to the TMH domain (Gensure et al., 2001; Assil-Kishawi and Abou-Samra, 2002; Dong and Miller, 2002; Dong et al., 2004). Most recently, strong corroborating evidence for the two-domain model has been obtained by the structural characterization of the isolated ECDs of several class B GPCRs (Grace et al., 2004; Parthier et al., 2007; Sun et al., 2007; Pioszak and Xu, 2008; Pioszak et al., 2008; Runge et al., 2008; Underwood et al., 2010).

## Molecular Evolution of GLP-1 and GLP1R for their Selective Interaction

Although GLP-1 and its paralogs share a high degree of sequence identity and structural similarly, they generally exhibit specific binding to their own cognate receptors with little cross-reactivity with paralogous receptors (Runge et al., 2003; Moon et al., 2010, 2012). This observation allows us to presume the presence of distinct amino acid residues within each peptide and receptor that allows for the selective interaction with their own partners. Further, these facts indicate that evolutionary selection pressure has exerted a specific diversification of peptide and receptor: variation among paralogs but conservation within orthologs. However, there are some exceptional cross-reactivities among paralogous partners. For instance, exendin, a GLP-1 paralog in non-mammals, has a high affinity for the mammalian GLP1R (Göke et al., 1993). Until now, genetic orthologs for GLP1R have not been found in teleost fish, yet teleost fish have two copies of GLP-1 peptides which have been generated by teleost-specific genome duplication (Plisetskaya and Mommsen, 1996; Irwin and Wong, 2005). Interestingly, GLP-1 is able to activate fish GCG receptor (GCGR) orthologs (Yeung et al., 2002; Irwin and Wong, 2005), indicating that fish GCGRs have achieved functional response to GLP-1 through an evolutionary process. These exceptional cross-reactivities provide a unique opportunity to explore the identification of specific amino acid residues in the peptides and receptors responsible for specific ligand-receptor interaction. For instance, amino acid sequence comparison between GLP-1 and exendin allows us to predict which amino acid residues are important for the activation of GLP1R (Moon et al., 2010). Sequence comparison between tetrapod GLP1Rs and fish GCGRs has led to the identification of residues in these receptors that interact with GLP-1 (Moon et al., 2012).

### Interaction between the α-helix of the peptide and the ECD of the receptor

The crystal structures of the ligand-bound ECD revealed that GLP-1 is a continuous α-helix from Thr^7^ to Val^27^, with a kink around Gly^16^. Only the residues between Ala^18^ and Val^27^ interact with the ECD. The α-helical segment of GLP-1 is amphiphilic, allowing hydrophilic and hydrophobic interactions through opposite faces of the α-helix (Underwood et al., 2010). The hydrophilic face of GLP-1 comprises residues Gln^17^, Lys^20^, Glu^21^, and Lys^28^, of which only Lys^20^ interacts directly with the ECD by forming a hydrogen bond with the side chain of Glu^128^. Interestingly, exendin also possesses a basic residue at position 20 (Arg^20^), allowing interaction with Glu^128^ of the GLP1R ECD (Runge et al., 2008; Underwood et al., 2010). The hydrophobic face of GLP-1 includes Ala^18^, Ala^19^, Phe^22^, Ile^23^, Leu^26^, and Val^27^. The hydrophobic residues are exposed toward the complementary hydrophobic binding pocket in the ECD. Particularly, Phe^22^, Ile^23^, and Leu^26^ of the peptide are found to interact with the highly conserved residues Val^36^, Trp^39^, Asp^67^, Tyr^69^, Arg^121^, and Leu^123^ in the ECD of GLP1R (Underwood et al., 2010). The contribution of Phe^22^, Ile^23^, and Leu^26^ to GLP1R binding has been demonstrated by Ala substitutions or mutations of these residues (Adelhorst et al., 1994; Wilmen et al., 1997). It is of interest to note that the GLP-1 family peptides exendin, GIP, GLP-2, and GCG also contain hydrophobic residues Phe^22^, Ile/Va^23^, and Leu^26^. Further, residues Trp^39^, Asp^67^, Tyr^69^, and Arg^121^ in the GLP1R ECD are also highly conserved in GLP2R, GIPR, and GCGR (Parthier et al., 2007; Runge et al., 2008; Underwood et al., 2010; Venneti and Hewage, 2011). This observation suggests that these residues are evolutionarily conserved and likely contribute to the primary binding between the α-helix of the peptides and ECD of the receptors. This may also in part account for the cross-interaction of one α-helix of the peptide with the ECD of other partners (Parthier et al., 2007; Runge et al., 2008; Underwood et al., 2010). Indeed, this is supported by the observations that chimeric peptides containing the GLP-1 N-terminus with the α-helix of GIP or GCG can induce GLP1R activation with a relatively high potency (Runge et al., 2003; Moon et al., 2010). However, the specific interaction of the α-helix of the peptide with the ECD of its own receptor may be of higher affinity than those with other related paralogous receptors. For instance, interaction of Ala^19^ of GLP-1 with GLP1R-specific Leu^32^, and Gln^19^ of GIP with GIPR-specific Ala^32^, may explain the higher affinity of each peptide toward its own receptor than toward paralogous receptors (Parthier et al., 2007; Underwood et al., 2010).

### Interactions between the N-terminus of the peptide and the core domain of the receptor

Specificity of ligand-receptor binding between a peptide and the corresponding receptor can be further conferred by binding between the N-terminus of the peptide and the receptor core domain. In addition, this interaction allows ligand-induced receptor activation (Thorens et al., 1993; Montrose-Rafizadeh et al., 1997; Hinke et al., 2001, 2003; During et al., 2003; Gault et al., 2007). Therefore, many approaches, such as alanine scanning, photoaffinity labeling, and molecular modeling-based approaches have explored the specific residues within the peptide and receptor responsible for ligand-receptor interaction (Adelhorst et al., 1994; Gallwitz et al., 1994; Xiao et al., 2000; Lopez de Maturana and Donnelly, 2002; Lopez de Maturana et al., 2004; Chen et al., 2009, 2010; Lin and Wang, 2009). Alanine scanning of GLP1R demonstrated that residues found between the TMH2 and ECL1 including Lys^197^, Asp^198^, Lys^202^, Met^204^, Tyr^205^, Asp^215^, and Arg^227^ are likely important for the binding of the receptor to the N-terminal moiety of GLP-1, as mutations at these residues lead to a significant decrease in ligand affinity (Xiao et al., 2000; Lopez de Maturana and Donnelly, 2002; Lopez de Maturana et al., 2004). However, these observations did not define how these individual residues interact with in the N-terminal moiety of GLP-1. Further, Ala mutations in these residues can modify the receptor conformation which may interfere with binding to the ligand. Recently, using photoaffinity labeling, Chen et al. (2010) observed that Tyr^205^ in ECL1 is in close proximity to the *p*-benzoyl-l-phenyl alanine (Bpa) at position 6 of GLP-1. However, the mutation of Tyr^205^ to Ala in GLP1R does not alter either receptor activity or ligand binding, indicating that Tyr^205^ is not the direct binding site for the N-terminal moiety of GLP-1. Furthermore, no ligand-bound crystal structure for the core domain of the class B GPCR family is currently available. Thus, our understanding of the molecular mechanism underlying the high affinity interaction between the N-terminal moiety of the peptide and the receptor core domain is primitive.

Recently, by comparing the amino acid sequences of GLP-1 and GLP1R with their orthologs and paralogs in vertebrates, we were able to obtain clues to help determine which amino acid residues may be responsible for ligand-receptor interaction (Moon et al., 2010, 2012). Although the GLP-1-related family of peptides shares a similarity in the amino acid sequence at the N-terminal moiety, there are specific, divergent amino acid sequences. For instance, the N-terminus of GLP-1 and its family peptides starts with either His^1^ (for GCG, GLP-1, GLP-2, and exendin) or Tyr^1^ (for GIP), and most of the peptides contain Thr at position 7 (GCG, GLP-1, and exendin), or Ser (GLP-2); only GIP contains Ile at this position. The first and seventh amino acid residues of each peptide are conserved within orthologs of vertebrate species. Thus, it may be postulated that His/Tyr^1^ or Thr/Ile^7^ residues of GLP-1 and GIP confer ligand selectivity to their cognate receptors. Indeed, our recent observation using chimeric GLP-1/GIP peptide reveals that the His/Tyr^1^ and Thr/Ile^7^ residues within these peptides confer differential ligand selectivity toward GIPR and GLP1R, respectively (Moon et al., 2010).

**Figure 3:**
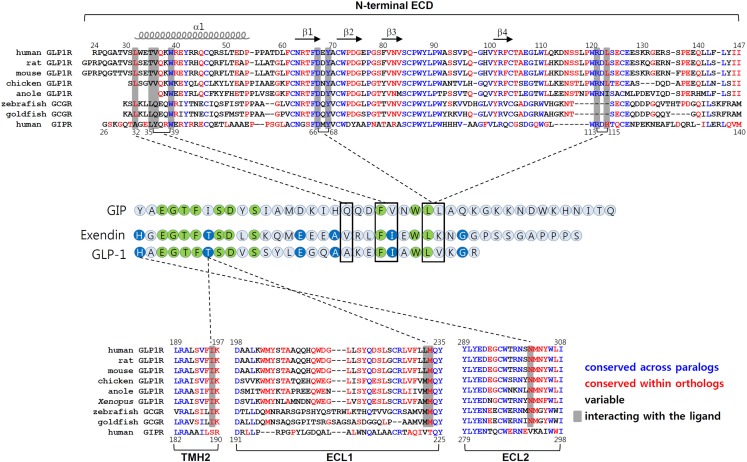
**Molecular interaction between GLP-1 and GLP1R**. The amino acid sequences of the N-terminal ECD, TMH2, ECL1, and ECL2 of vertebrate GLP1Rs, fish GCGRs (which are known to bind GLP-1), and human GIPR are shown in top and bottom panels. The residues in blue are conserved between GLP1Rs and GIPR. Residues shown in red are conserved sequences within the orthologous receptor of vertebrates. The residues in black are variable sequences. The amino acid residues that interact with their peptides are shaded. Amino acid sequence alignment of GLP-1, exendin, and GIP are shown in the middle. Residues which are identical among three peptides are in green. Residues common for GLP-1 and exendin are in blue. The interaction between peptides and receptors are indicated by dotted lines.

The chimeric GLP1R/GIPR approach together with chimeric GLP-1/GIP peptides offers a new strategy to determine which residues in the core domain are responsible for interacting with His^1^ and Thr^7^ of GLP-1 (Moon et al., 2012). For example, this approach enables us to determine crude motifs in TMH2, ECL1, and ECL2 of GLP1R which are likely to interact with His^1^ and Thr^7^ of GLP-1. Amino acid sequence comparison of these regions between those of tetrapod GLP1Rs, fish GCGRs, and vertebrate GIPRs further define amino acid residues that tentatively interact with His^1^ and Thr^7^ of GLP-1. In this case, we searched for residues which are conserved within GLP1R ortholog and fish GCGR but are different from those of GIPRs. We were able to identify Ile^196^ and Lys^197^ of TMH2, and Met^233^ of ECL1, and Asn^302^ and Met^303^ of ECL2 in GLP1R (Moon et al., 2012). It is noteworthy that fish GCGRs exhibit both a significantly high affinity for GLP-1 (Irwin and Wong, 2005) and these conserved residues, even though other regions are significantly different from tetrapod GLP1Rs (Figure 3). Mutational mapping together with application of chimeric GLP-1/GIP peptides reveals that His^1^-harboring peptides are sensitive for Asn^302^ mutations, while Thr^7^-containing chimeric peptides are highly sensitive for the Ile^196^ mutation, indicating a possible interaction of His^1^ and Thr^7^ of GLP-1 with Asn^302^ and Ile^196^ of GLP1R, respectively. Indeed, computer-aided molecular modeling showed interaction of His^1^ with Asn^302^ and of Thr^7^ with a binding pocket formed by Ile^196^, Leu^232^, and Met^233^ of GLP1R (Moon et al., 2012). Interestingly, Asn^302^ is highly conserved at the corresponding position in GCGRs (Asn^300^ for human) and GLP2Rs (Asn^336^ for human), and these receptors have peptide ligands containing His^1^. In addition, GCGRs that respond to a Thr^7^-containing peptide ligands have conserved Val^193^ and Met^231^ at the corresponding positions of Ile^196^, and Met^233^ of GLP1R, indicating that these residues may have a contact with Thr^7^ of GCG. This possibility, however, needs to be further addressed.

## Conclusion

Although GLP-1 may become a promising therapeutic agent for the treatment of type 2 diabetes mellitus and obesity, these peptide agonists cannot be administered orally due to their peptide nature. Thus, orally administered small molecules that regulate GLP1R need to be developed. However, a bottleneck slowing the development of these small molecules is the lack of information regarding the molecular structure of the ligand-bound GLP1R. Unfortunately, the crystal structure of the ligand-bound N-terminal ECD of GLP1R may not fully account for the interaction between peptide ligands and receptors. Rather, it is likely that the seven TMHs and ECLs of the receptors are more critical than the N-terminal ECD for peptide binding and receptor activation. Thus, exploring the domains or amino acid residues within TMHs and ECLs that confer ligand binding and receptor activation may greatly contribute to the design of the molecular model for the peptide ligand-receptor complex. In turn, this would facilitate the development of potent small molecules capable of regulating GLP1R. Determination of ligand-receptor interaction points (either by ligand-bound ECD crystal structure or by biochemical analysis using chimeric peptides and receptors) demonstrates that conserved residues across paralogous peptides tend to interact with conserved residues among the paralogous receptors while the same can be said of conserved residues within orthologous peptides that likely interact with conserved residues within orthologous receptors. Thus, evolutionary conservation across ortholog and specific diversification within paralogs may be required for maintaining the selective interaction of a peptide ligand with its cognate receptor. The crystal structure of ligand-bound GLP1R ECD explains how residues between Ala^18^ and Val^27^ interact with the ECD. Biochemical studies using chimeric peptides and receptors reveal receptor contact points only at His^1^ and Thr^7^. Thus, the residues between the N-terminus through to position 17 of GLP-1 remain to be explored for their impact on binding to the receptor. Comparative biochemical approaches combined with computer-aided molecular modeling will discover additional residues in the peptide and receptor sequences that help understanding the high affinity binding interaction between the ligand-receptor pair.

## Conflict of Interest Statement

The authors declare that the research was conducted in the absence of any commercial or financial relationships that could be construed as a potential conflict of interest.

## Abbreviations

Bpa, *p*-benzoyl-l-phenyl alanine; CRFR, CRF receptor; Cys, cysteine; ECD, extracellular domain; ECL, extracellular loop; ER, endoplasmic reticulum; GCG, glucagon; GCGR, glucagon receptor; GIP, glucose-dependent insulinotropic polypeptide; GIPR, GIP receptor; GLP-1, glucagon-like peptide-1; GLP1R, GLP-1 receptor; GPCR, G protein-coupled receptor; PAC1R, PACAP receptor; PTHR, PTH receptor; TMH, transmembrane helix.
